# Listening to Communities: Mixed-Method Study of the Engagement of Disadvantaged Mothers and Pregnant Women With Digital Health Technologies

**DOI:** 10.2196/jmir.7736

**Published:** 2017-07-05

**Authors:** Sylvia Guendelman, Andrew Broderick, Hmellisa Mlo, Alison Gemmill, David Lindeman

**Affiliations:** ^1^ Maternal and Child Health School of Public Health University of California, Berkeley Berkeley, CA United States; ^2^ Center for Innovation and Technology in Public Health Public Health Institute Oakland, CA United States; ^3^ Department of Demography University of California, Berkeley Berkeley, CA United States; ^4^ CITRIS Health Initiative The Center for Information Technology Research in the Interest of Society (CITRIS) and the Banatao Institute Berkeley, CA United States

**Keywords:** digital divide, health information management, consumer health information, pregnant women, mothers

## Abstract

**Background:**

US health care providers are increasingly demanding patient engagement with digital health technologies to enroll in care, access personal health information, communicate with providers, and monitor their own health. Such engagement may be difficult for disadvantaged populations who may have limited health literacy, time constraints, or competing priorities.

**Objective:**

We aimed to understand the extent of adoption and use of digital health tools and to identify key perceived psychological motivators of technology use among disadvantaged first-time pregnant women and mothers of young children.

**Methods:**

We recruited women from health organizations serving low-income communities in the Midwest and on the East and West coasts. A total of 92 women participated in 14 focus groups. During each session, we administered worksheets that measured 3 utilization outcomes: the number of recent Web-based health-seeking activities, current use of digital health-management practices (eg, accessing personal health information, communicating with providers, and scheduling appointments), and potential adoption of digital health-management tools among low users or nonusers. Responses to the worksheets and to a pre-focus group survey on demographics, technology access, and motivators of use were examined to create user profiles. Separate regression models identified the motivators (eHealth literacy, internal health orientation, and trust in digital information) associated with these outcomes. Qualitative data were incorporated to illustrate the worksheet responses.

**Results:**

Whereas 97% of the participants reported that they had searched for health information on the Internet in the past year, 42% did not engage in digital health-management practices. Among the low users and nonusers, 49% expressed interest in future adoption of digital health tools. Web-based health information-seeking activities were associated with digital health-management practices (*P*<.001). When controlling for covariates, eHealth literacy was positively correlated with the number of Web-based health-seeking activities (beta=.03, 95% CI 0.00-0.07). However, an internal health orientation was a much stronger correlate of digital health-management practices (beta=.13, 95% CI 0.02-0.24), whereas trust in digital information increased the odds of potential adoption (vs no adoption) in adjusted models (OR 5.21, 95% CI 0.84-32.53). Demographic characteristics were not important drivers of digital health use and few differences distinguished use among mothers and pregnant women.

**Conclusions:**

Seeking health information on the Internet may be an important gateway toward engaging in digital health-management practices. Notably, different consumer motivators influence digital health tool use. The relative contributions of each must be explored to design tools and interventions that enhance competencies for the management of self and child health among disadvantaged mothers and pregnant women. Unless we address disparities in digital health tool use, benefits from their use will accrue predominantly to individuals with the resources and skills to use technology effectively.

## Introduction

Health care providers are increasingly implementing applications that demand patient engagement with digital technologies to enroll in care, mediate the use of electronic health records, communicate with their providers, and monitor their own care. Evidence shows that health care is more efficient and effective when patients are actively involved in their own treatment [1]. Engaged patients who collaborate with their providers are more likely to be treated with respect, receive information related to their care, and become involved in their health care decisions [2,3]. Furthermore, active information seekers are more likely to engage in preventive health behaviors such as physical activity and healthy dietary behaviors [4].

The digitalization and quantification of health care has proven to be difficult for disadvantaged populations who may have limited health literacy, time resources, and competing priorities [5-7]. Several studies have shown that individuals with a lower socio-economic status and of non-white race or Hispanic ethnicity are less likely than their more affluent, white counterparts to engage in Internet health-seeking behaviors [8,9], although results documenting these disparities have been inconsistent [10,11]. Evidence further demonstrates that Web-based health search activities among adults are more common than other digital health practices that involve personal health-management or caregiving behaviors [8,12]. A recent review showed that patients’ interest and ability in using patient portals is strongly influenced by demographic factors (ie, age, ethnicity, and education), health literacy, health status, and caregiver roles [13]. Yet, many applications for personal digital health management have been created with a “design it and they will come” approach that may not be appropriate or meaningful for use by individuals whose health literacy, cultural values, or trust limits their ability or willingness to use digital tools [14]. Whereas the evidence on the use of digital health technology derives mainly from the general population of adults, seniors, and adolescents, or patients with chronic diseases, information on the drivers of use by specific low-income subpopulations such as pregnant women or mothers of young children is sparse [15].

Evidence indicates that first-time pregnant women or those caring for their first infant are particularly likely to use digital health technology as they have a stronger need and desire to acquire pregnancy and child health information and seek social support [16,17]. Some women turn to digital media to compensate for the lack of information or support provided during prenatal visits [18]. Others, who disagree with the information provided to them by health professionals or perceive a lack of time to ask questions, use the Internet to bridge information gaps [19]. The overwhelming amount of Web-based information that requires women to assess what information to trust, the time and confidence required to find appropriate information, the lack of interest in evidence-based information, and feelings that more information would not help make more informed medical decisions are some identified information-seeking barriers among pregnant women [15,20].

It is unclear whether the information needs, skills, and barriers found among pregnant women are similar to those of mothers of young children. A recent study of pregnant women showed that self-efficacy and internal health locus of control contributed to health information-seeking [11], whereas others report that health information-seeking on the Internet remains less trustworthy when compared with doctors, friends, and family [15,21]. Previous studies have mainly focused on the relationship of eHealth literacy and health information-seeking on the Web and far less is known about how different motivational factors contribute to the use of digital tools for health-management purposes or digital tool adoption among pregnant women and mothers of young children [15].

To help design interventions and consumer-centered tools that improve access to and use of health services among low-income first-time pregnant women and mothers of young children, this study aimed to understand the extent of adoption and use of digital health tools and to identify key perceived psychological motivators of technology use.

## Methods

This mixed-methods study conducted community engagement listening sessions involving focus groups with first-time pregnant women and mothers of children under the age of five. The study was designed to assess how participants experience and use technology for their health or their children’s health and how tools such as websites, apps, wearables, social networks, video chats, and patient portals could be used to better meet their needs in managing health in the future. Each focus group session included three brief worksheets that quantified participants’ recent health-related search activities on the Internet, their current use of digital technologies to support their health management, and interest in adopting these technologies in the future, respectively. The focus group guide amplified the information sought in these worksheets, assessed their use preferences, and identified key motivators and barriers to health technology use. Responses to the worksheets and to a pre-focus group survey on sociodemographic characteristics, technology ownership and access, eHealth literacy, and personal agency were used to create a profile of users and to identify determinants of use.

### Sample

Study participants were recruited from community clinics, federally qualified health centers, Women, Infants, and Children (WIC) clinics, and nongovernmental organizations serving low income communities in the San Francisco (SF) Bay Area, New York’s South Bronx district, and West Louisville, Kentucky. Active recruitment by clinic providers and staff, email invitations, and flyers posted in the potential sites were used to encourage participation. A few women were also recruited by study participants. Recruitment materials invited women to participate in a focus group to share their opinions about their experience with technology for health and well-being. Eligibility criteria included being 18 years old or older, currently pregnant or a mother of a young child, residing in the study area, and being able to read, write, and speak English. The study was approved by the UC Berkeley Committee for the Protection of Human Subjects (ID: 2016-06-8837).

### Procedures

Between June and November 2016, we conducted 14 focus groups, ranging from 2 to 14 participants each, with a total of 92 participants. Focus group sessions, including 2 in the Bronx, 2 in Louisville, and 10 in the SF Bay Area were held at the recruiting sites or a nearby community center; each lasted approximately 2 hours. Sessions were facilitated by one investigator trained in qualitative methods (AB), with the help of two others (HM, SG) who actively greeted participants, distributed the pre-focus group survey and worksheets and took notes. Informed consent was obtained prior to beginning the focus group sessions. Compensation between US $20 and $35, depending on the study site, was offered to the participants upon completion of the focus group. Stipends for childcare and transportation were provided at some sites. All sessions were audiotaped and transcribed.

### Quantitative Measures

Three primary outcome variables measured the use of health technologies *.*

#### Number of Internet Health-Seeking Activities

This outcome was defined as the number of health-related categories searched on the Internet in the past 12 months, which included information about a specific disease or medical problem, a drug, medical treatment, test or procedure, safety concerns, pregnancy and childbirth, diet and weight, breastfeeding, caregiving, and health insurance issues. This variable was restricted to search users and treated as a continuous variable for modeling purposes and as a categorical three-part variable (<5, 5-8, 9+) for descriptive purposes, based on the median and the 75th percentile of the distribution of total number of search activities (Median=5; 75th%=8).

#### Current Use of Digital Health-Management Practices

This outcome was assessed by asking participants to identify from a list the practices they used, such as accessing medical information or scheduling appointments through patient portals, communicating with providers through secure email messaging or video chats, managing or tracking their own health or their children’s health with apps or wearables, receiving text message appointment or medication reminders or health education, writing Web reviews of medical treatments or providers, or engaging with social networks or patient groups for health-related reasons on the Internet. Users were categorized as high users or adopters if they engaged with digital technology tools in 4 or more health-management practices, which put them in the 75th percentile or above, low users if they engaged in 1 to 3 practices with technology tools, and nonusers if they reported no current use of digital health tools. The total number of digital health-management practices used was treated as a continuous variable for modeling purposes.

#### Potential Adopters

Potential adopters were those who were highly interested in using digital technology for specific health-management practices in the future, but were currently either nonusers or low users. Interest was gauged by asking the extent to which participants were interested in engaging in different practices, such as receiving text message appointment reminders, by using a 4-point Likert scale (not at all interested, moderately interested, very interested, extremely interested). Participants who stated that they were not at all interested in 2 or more practices were classified as having low interest. Potential adopters were compared with high users and non-adopters.

Independent variables consisted of 3 motivational factors that were amenable to change, namely eHealth literacy, internalized health orientation, and satisfaction with the trust in digital information. eHealth literacy was measured using the eHealth Literacy Scale (eHEALS), an eight-item self-reported measure of perceived eHealth literacy. The tool provides an estimate of an individual’s combined knowledge, confidence, and perceived skill at finding, evaluating, and applying electronic health information to health problems [10]. Based on a 5-point Likert-type scale (1=strongly disagree and 5=strongly agree) participants indicate their level of agreement with eHealth statements, with higher scores indicating higher eHealth literacy. Score totals ranged from 8 to 40. The reported Cronbach alpha coefficient of the tool in our study is .88, which is similar to that in other studies: .88 [22] and .89 [10].

Internal health orientation refers to an individual’s motivation to engage in healthy attitudes, beliefs, and behaviors [23], and this study was based on an index of three consumer orientations: *“I am responsible for my own health,”* *“I am actively taking care of my health,”* and *“I should be in control of who has access to my health data.”* Participants were asked in the pre-focus group survey to indicate their level of agreement with these statements on a 5-point Likert-type scale (1=strongly disagree and 5=strongly agree). Score totals ranged from 3 to 15, with a Cronbach alpha value of .67. A Cronbach alpha value of .65 has been suggested as a minimum acceptable value [24].

Trust in digital information was a categorical measure of whether users of technology were satisfied (“Yes”) or were unsure or dissatisfied (“No”), with the trust placed on the information obtained from digital sources such as websites or videos.

Demographic variables, health status, and computer or smartphone use or ownership were included as potential covariates. Demographic characteristics included childbearing status, geographic location (Bronx, Louisville, or SF Bay Area), race or ethnicity, marital status, educational level, employment status, and health insurance type. We used self-rated health (whether excellent, very good, good, or fair or poor) as our measure of health.

### Data Analysis

Stata version 14.1 (StataCorp LP) was employed to analyze the quantitative data. Univariate analysis was used to characterize the study population and bivariate analyses using chi-square tests for categorical variables and one-way analysis of variances (ANOVAs) for continuous variables were performed to examine the associations between demographic characteristics and motivational factors and the primary outcomes. Given the small sample size (n=92), we consider *P* values ≤.10 as significant. Separate regression models were estimated to determine which variables were related to each of the 3 primary outcomes. Linear regression was used to identify the main correlates of the total number of Web-based health-seeking activities and the total number of digital health-management practices. Logistic regression was used to identify the associations of the motivational factors with potential adoption, no interest in adoption, and high adopter subgroups. The results of the regression models are presented as beta coefficients or odds ratios (ORs) and 95% CIs. Furthermore, by taking the log of each continuous outcome, we estimated the percentage change in the outcome with each unit change in the independent variable by exponentiating the beta coefficient. Our models first estimated the unadjusted risk of each motivational factor (Model 1) and then the adjusted risk of each factor controlling for the two other motivational factors as well as childbearing status, marital status, education, geographic location, and self-rated health (Model 2). Whereas childbearing status was forced in, the remaining demographic and health covariates were selected because they were associated with at least one of the primary outcomes or with at least one of the motivational factors (*P* ≤.10). All models adjusted for the same covariates.

Subsequently, two members of the research team (SG and HM) independently analyzed each focus group transcript to further understand the recurring themes or those that were discussed most or least extensively regarding each of the technology use outcomes identified in the quantitative analysis. To ensure analytic rigor, several verification strategies were applied, including multiple readings of the transcripts, iterative generation of themes, and checking against all focus group transcripts to assess the extent to which they were shared by participants. Illustrative examples of the themes were selected and presented.

## Results

### Characteristics of Study Participants and Engagement With Digital Health Technologies

More than 1 out of 4 participants (28%) were pregnant for the first time and 72% were mothers of young children (see Multimedia Appendix 1). The majority were between 25 and 34 years old, black or Hispanic, married or cohabitating with their partners, had attained some college education, were either unemployed or not in the labor force, on Medicaid (MediCal in California) and rated their health as good or very good (Multimedia Appendix 1). For the majority, housing and getting or holding a job, rather than health, were their primary reported life concerns (data not shown).

Most had access to technology—84% owned or had access to a computer and 87% owned or used a smartphone and this access was correlated with Web searching (Multimedia Appendix 1). Only 3% of the participants reported that they had not used the Internet to search for health information in the last 12 months. The majority resorted to Google searches, although YouTube, Facebook, and Yahoo were also mentioned. Among Internet users, 25% engaged in a high number of Web-based health search activities, usually with confidence and precision; 38% engaged in 5 to 8 search activities, whereas 37% reported that they had engaged in very few Web-based search activities, applied no particular search strategies, and did not want to delve deeper into information because it can be overwhelming or confusing:

I usually go to WebMD and Baby Center. Sometimes the information is useful. When I go to another website, and it say something way different than other websites, I don’t know what to choose.

Use of digital health-management practices was low; approximately 42% and 30% of the study participants reported no current use or low current use, respectively. Many mentioned that they preferred face-to-face contact with providers or with other mothers to seek and share information, advice, and support. Others expressed a strong need to claim their personal space:

I don’t use social media. I like keeping things to myself and for just the people I know.

About half of the current nonusers or low current users of digital health-management practices expressed little interest or intention to use patient portals, text reminders, or text messaging to connect with providers:

I signed up to use a portal, but I never used it. I forgot about it...I just prefer calling and visiting the center. When it comes to my health, I’d rather come and talk to someone in person and same for my child.

I’m slightly interested in My Chart but I’m not trippin’ about it because my daughter’s nurse comes to the house...and I trust the nurse because I can see what she is doing.

However, among the current nonusers or low current users of digital health-management practices, 49% were classified as potential adopters because they expressed high interest in engaging with digital health-management tools in the future. Some potential adopters were already using the patient portal, but infrequently or for one specific purpose, such as scheduling appointments, emailing doctors, or getting text reminders. Several mentioned that they would like to use the portal, but had not been taught how to do so:

I’m interested in connecting more with my doctor and my kids’ doctor, but who is there to help me do it? If we don’t have time to sign up and they don’t have time to help us, then I won’t do it.

Although potential adopters mentioned that they relied on Internet searches (mainly Google) and apps like Baby Center, they often preferred TV (Dr. Oz), books, and face-to-face encounters with providers:

When I was first pregnant, I searched for a lot of apps because I wanted to know everything. But mostly, people just talk about their concerns online...I just call my advice nurse.

Only 27% were high users of digital health-management practices for their own or their children’s health. They mostly used a variety of apps such as What to Expect, Bump, Baby Center, and fitness and ovulation apps:

I use pregnancy apps and get updates everyday like how big my baby is this week. There’s also a community part that I use sometimes to talk to other [pregnant] women who are experiencing the same things I am. I watched a lot of pregnancies on YouTube...it’s neat. I tried to sign up for insurance online, but kept getting road blocked.

High users of digital health-management practices also tended to interact with the patient portal and liked the multiple functions it offers:

I like that I’ve been able to see exactly how things over time have happened [in the portal].

I find the portal useful. I might not be able to make it in person because of transportation issues or I might not have my phone on. It’s an alternative to contacting my doctor without having to sit and wait. It’s easier to get messages through.

Participants’ number of Internet search activities, current use of digital health-management practices, and intention to use digital health-management tools did not vary significantly by demographic characteristics, with the exception of geographic location (Multimedia Appendix 1). However, a higher proportion of mothers than first-time pregnant women engaged in a higher number of Internet search activities (*P*=.10) *.* Educational level was associated with both high and low use of digital technology for health-management practices (*P*=.05) and number of Internet search activities (*P*=.10). For instance, proportionately more women with some college education, but fewer with a bachelor’s degree or higher qualification, were current high users of digital health-management practices. By contrast, women with incomplete or no high school education were the most likely to not engage in digital health-management practices. Furthermore, a higher proportion of married or cohabiting women reported interest in adopting digital health technology (*P*=.05).

### Motivational Drivers of Digital Health Technology Use

#### Number of Internet Health-Seeking Activities

The number of Internet health-seeking activities in the past 12 months was positively and significantly associated with the eHEALS score (Table 1). For every unit increase in eHEALS, the number of searches increased by 3% (beta=.03, 95% CI 0.00-0.06). This relationship, although marginally significant, persisted (beta=.03, 95% CI 0.00-0.07), reflecting a 3% change for every unit increase in eHEALS when adjusting for internal health orientation, trust in digital information, and other demographic covariates (ie, childbearing status, marital status, education, geographic location, and self-rated health).

#### Current Use of Digital Health-Management Practices

Current use of digital health-management practices (no use, low use, or high use) was significantly associated with the number of search activities (*P*<.001) (data not shown). Nonetheless, it was not significantly associated with eHEALS scores (Table 2). Whereas the total number of digital health-management practices was marginally correlated with eHEALS scores in unadjusted models (beta=.03, 95% CI 0.00-0.07), this correlation was no longer significant after adjusting for the covariates shown in Table 2. In contrast, internal health orientation scores were positively and significantly correlated with the total number of digital health-management practices in both unadjusted (beta=.12, 95% CI 0.02-0.22) and adjusted models (beta=.13, 95% CI 0.02-0.24), such that for every unit increase in scores, the total number of digital health-management practices increased by 14%. Trust in digital information was associated both with the current level of use (*P*=.05) and the total number of digital health-management practices (beta=.51, 95% CI 0.05-0.96), *P*=.05, but was no longer significantly correlated with the total number of digital health-management practices when controlling for other covariates.

**Table 1:** Associations between number of Internet search activities in the last 12 months and eHealth literacy (eHEALS), internal orientation toward health, and trust in digital information.

Motivational factor	Number of Internet search activities in the last 12 months			Model 1		Model 2^d^	
	<5	5-8	9+	beta	95% CI	beta	95% CI
	Mean or n (%)	Mean or n (%)	Mean or n (%)				
eHEALS score, mean	29.6^a^	32.4^a^	33.2^a^	.03^a^	0.00-0.06	.03^b^	−0.00 to 0.07
Internal orientation toward health score, mean	13.5	13.7	13.7	.01	−0.08 to 0.10	.02	−0.07 to 0.12
Trust in digital information^c^, n (%)	23 (69.7)	30 (88.2)	18 (85.7)	.23	−0.18 to 0.64	.16	−0.28 to 0.60

^a^*P*=.05.

^b^*P*=.10.

^c^Reference category in linear regression models: not satisfied or neutral about trust in digital information.

^d^Model 2 adjusts for all variables shown in the table as well as the following covariates: childbearing status, marital status, education, geographic location, and self-rated health.

**Table 2:** Associations between current use of digital health-management practices and eHEALS, internal orientation toward health, and trust in digital information.

Motivational factor	Current use of digital health-management practices			Model 1		Model 2^d^	
	No use	Low use	High use	beta	95% CI	beta	95% CI
	Mean or n (%)	Mean or n (%)	Mean or n (%)				
eHEALS, mean	30.1	32.4	32.1	.03^a^	−0.00 to 0.07	.01	−0.03 to 0.05
Internal orientation toward health, mean	13.2^a^	13.4^a^	14.2^a^	.12^b^	0.02-0.22	.13^b^	0.02-0.24
Trust in digital information^c^, n (%)	23 (70.3)^b^	22 (78.6)^b^	24 (96.0)^b^	.51^b^	0.05-0.96	.31	−0.21 to 0.83

^a^*P*=.10.

^b^*P*=.05.

^c^Reference category in linear regression models: not satisfied or neutral about trust in digital information.

^d^Model 2 adjusts for all variables shown in the table as well as the following covariates: childbearing status, marital status, education, geographic location, and self-rated health.

#### High Adopters Versus Potential Adopters

High adopters had higher mean internal health orientation scores than potential adopters or those who lacked interest in adopting technologies in the future (14.2 vs 13.1 vs 13.7, respectively), with *P*=.05 (Tables 3 and 4). A higher internal health orientation more than tripled the odds of becoming a high adopter versus a potential adopter in adjusted models (OR 3.13 95% CI 1.26-7.78). Additionally, a higher proportion of high adopters reported having trust in digital information as compared with potential adopters or with those who lacked interest in adopting technologies (96% vs 81% vs 59%, respectively, with *P*=.01). Whereas the odds of high adoption versus potential adoption were not significantly associated with trust in digital information, the odds of potential adoption versus no adoption were 3 times higher among women who trusted the health information found from digital health sources compared with those who did not trust the information. The odds of potential adoption were even higher and marginally significant when adjusting for demographic covariates and the other motivational factors (OR 5.21, 95% CI 0.84-32.53). Potential adopters stated that they were “extremely interested” in accessing a repository for all their health-related information, engaging in secure email messaging with their physicians, getting text messages for appointment reminders, and being able to map local community resources such as housing and childcare (data not shown).

**Table 3:** Associations between intention to use digital health-management tools and eHEALS, internal orientation toward health, and trust in digital information for potential adopters versus no or low interest.

Motivational factor	Intention to use digital health-management tools		Potential adopter versus no interest or low interest			
	No interest or low interest	Potential adopter	Model 1		Model 2^e^	
	Mean or n (%)	Mean or n (%)	OR	95% CI	OR	95% CI
eHEALS, mean	30.4	31.5	1.04	0.94-1.15	1.03	0.86-1.23
Internal orientation towards health, mean	13.7^a^	13.1^a^	0.82	0.58-1.16	0.58	0.28-1.22
Trust in digital information^d^, n (%)	13 (59.1)^b^	35 (81.4)^b^	3.03^c^	0.96-9.52	5.21^c^	0.84-32.53

^a^*P*=.05.

^b^*P*<.001.

^c^*P*=.10.

^d^Reference category in linear regression models: not satisfied or neutral about trust in digital information.

^e^Model 2 adjusts for all variables shown in the table as well as the following covariates: childbearing status, marital status, education, geographic location, and self-rated health.

**Table 4:** Associations between intention to use digital health-management tools and eHEALS, internal orientation toward health, and trust in digital information for high interest versus potential adopters.

Motivational factor	Intention to use digital health-management tools		High adopter versus potential adopter			
	Potential adopter	High adopter	Model 1		Model 2^e^	
	Mean or n (%)	Mean or n (%)	OR	95% CI	OR	95% CI
eHEALS, mean	31.5	32.1	1.02	0.93-1.12	0.92	0.75-1.14
Internal orientation towards health, mean	13.1^a^	14.2^a^	1.59^a^	1.07-2.37	3.13^a^	1.26-7.78
Trust in digital information^d^, n (%)	35 (81.4)^b^	24 (96.0)^b^	5.49	0.64-46.75	2.55	0.04-164.47

^a^*P*=.05.

^b^*P*<.001.

^c^*P*=.10.

^d^Reference category in linear regression models: not satisfied or neutral about trust in digital information.

^e^Model 2 adjusts for all variables shown in the table as well as the following covariates: childbearing status, marital status, education, geographic location, and self-rated health.

## Discussion

### Principal Findings

Among the low-income pregnant women and mothers of young children who participated in this study, we found very high access to smartphones and computers which, as expected, was positively correlated with use of the Internet for health information-seeking. Whereas 97% of participants reported that they had searched the Internet for health information in the past year, 25% reported that they had conducted a high number of search activities, despite health concerns not being their highest priority in life. These proportions are much higher as compared with the overall proportion of adult women who reported that they searched for health information on the Internet. A previous study using a nationally representative sample from the National Health Interview Survey found that between 2009 and 2013, 50% of women had used the Internet for health information-seeking [25]. A Pew Research Center study found that in 2013, 62% of Americans had looked for health information on the Internet within the past year [26].

When compared with other Web-based searches, we found a much lower use of the Internet or other digital tools for health-management practices such as for accessing personal health information or scheduling appointments through patient portals, communicating with providers through secure email messaging or video chats, use of health tracking apps or wearables, or engaging with social networks or patient groups on the Internet. Only 27% of participants engaged in 4 or more digital health-management practices, whereas 42% engaged in none. Previous studies by Pew and others have confirmed this disparity in functional use in non-pregnant populations [8,12].

Notably, Internet health information-seeking behaviors were closely associated with digital media use for health management, suggesting that health information-seeking may be an important gateway toward using digital health-management practices. As a somewhat larger percentage of mothers of young children than first-time pregnant women engaged in Internet search activities, pregnant women’s Internet use should be considered an important target for intervention.

The number of Internet search activities was positively correlated with eHEALS scores, even after controlling for the two other motivational factors, demographic variables, and health status. Digital health literacy has been identified as an important driver of health technology usage in other studies [10,27]. Healthy People 2020 goals strive to increase health literacy skills and recognize the influence of health literacy on health status and the quality of care [28]. However, as the findings of this study show, eHealth literacy was a weaker predictor of the total number of digital health-management practices or of potential adoption of digital health tools, indicating that other motivational factors are more important drivers of these outcomes. Specifically, we found that an internal health orientation was a strong and significant correlate of total number of digital health-management practices, whereas trust in digital information increased the odds of potential adoption (vs no adoption) of digital health technology, after controlling for other variables in our models. Two previous studies have shown that individuals with a high consumer orientation or internal locus of control have a higher motivation to search and comprehend health information [11] and to adopt digital health [29]. A recent systematic review of qualitative studies on consumer engagement with digital health also found that personal agency over one’s health was associated with digital tool use [27]. According to Song and colleagues [30], our current health care system values the informed patient who is “responsible, self-aware, vigilant and savvy” and personal agency helps to actualize these norms.

Whereas prior research on digital health use has focused predominantly on the role of eHealth literacy, other motivational factors have received less attention. Future studies using prospective designs with larger samples of first-time pregnant women and mothers of young children could shed further light on the links between internal consumer orientations, trust, and digital engagement. A better understanding of these associations could lead to the development of better tools and higher consumer engagement.

Demographic characteristics were not important drivers of digital health use in this study population. Whereas geographic location was associated with the outcomes in the bivariate analyses, it was not a significant predictor of the outcomes in the multivariate models. A 2015 nationally representative survey of digital health adoption conducted by Rock Health also found that demographics was not associated with digital health adoption, whereas a consumer orientation, based on similar beliefs as those examined in our study, had a robust relationship with digital health adoption [29]. We also found that first-time pregnant mothers did not differ significantly from mothers of young children in their current use of digital health-management practices or potential adoption of digital health tools.

Competent health communication and proficient use of health information technology are considered essential attributes of an informed consumer. Yet, many health programs aimed at engaging patients through technology struggle to reach underserved populations. Improving engagement with digital health among vulnerable pregnant women and mothers may require the following actionable steps: (1) fostering provider encouragement of Internet health seeking information, (2) encouraging providers to query patients about their Internet search behaviors, (3) enabling trainings to increase public awareness of different digital health tools, and (4) bolstering women’s personal agency.

Firstly, provider encouragement of Internet health-seeking information must be fostered. Web searching may be an important gateway toward active management of women’s own health or that of their children and may help bolster women’s roles as active informed patients. Similar to a previous systematic review, we found that clinical endorsement from trusted providers enhances consumer engagement [27].

Secondly, providers should be encouraged to query patients about their Internet search behaviors. Assessing consumers’ comfort in using tools that require eHealth allows for the identification of skill gaps and interests in adopting digital technologies.

Thirdly, trainings should be conducted to increase public awareness of different digital health tools (including patient portals). The working of these tools should be explained and the potential benefits and risks to safety, security, and sense of privacy should be identified. Trainings should build skills, confidence, and trust in the use of these tools, and they could be targeted at consumers and providers.

Finally, women’s personal agency should be bolstered so that they can confidently assume that they are responsible for and can influence their own and their child’s health. Many women already use social support groups and express interest in Web-based services that are localized, social, and link to community resources.

Failure to address the disparities in digital use found in this study suggests that the benefits from the use of digital health solutions will accrue only to those individuals with the resources and skills to use technology effectively. This could exacerbate inequities in already vulnerable populations. Strategies to eliminate digital health inequities could benefit from further research on the drop off in the number of users in the transition from Internet searches to digital health-management practices. The findings from such research would further inform strategies for designing interventions that promote adoption and routine use of digital health-management practices and patient interactions with the health care system.

### Limitations

This study has several limitations. We used a small convenience sample of low income, English-proficient, urban dwellers, which does not allow us to generalize the findings to other pregnant women or mothers. We mostly recruited publicly insured women enrolled in primary care clinics and other programs. Evidence indicates that individuals who experience difficulties in accessing health care for reasons unrelated to their insurance status are more likely to report using the Internet for health information [5]. We relied on self-reports and measures that corresponded to perceived skills and attributes, not to actual skills, knowledge, motivation to use digital health-management tools, or adoption of and engagement with digital health technologies. Furthermore, our cross-sectional study design does not allow us to assess temporal or causal relationships. Further research with prospective or experimental designs is needed to corroborate our findings.

Our study also had strengths. We restricted the study population to pregnant women and mothers of young children, allowing us to focus on an important life stage, which presents unique opportunities for behavior change and adoption of digital health technology. Unlike many studies that have focused on a particular patient population in a specific setting and a single technology [27], we sampled a diverse group of participants from various clinics and programs from communities in several geographic locations. Furthermore, we expanded our scope beyond Internet health searches to include use of digital health technology for health management and used a mixed-methods approach to gather information.

### Conclusions

This study showed that Web-based health information searches were widespread, whereas use of digital health-management practices was far less common in this sample of low-income, first-time pregnant women and mothers. The results demonstrate a significant relationship between Internet health search activities and engagement in digital health-management practices. Whereas higher eHealth literacy was strongly associated with Web search activities, an internal health orientation was more strongly associated with the number of digital health-management practices, and trust in digital information was associated with potential adoption of digital tools. The relative contributions of these consumer motivations for use of digital health technologies need to be further explored to design better tools and interventions that address this population’s interests and enhance the competencies to manage self and child health.

## Appendix

Multimedia Appendix 1 Demographic, health, and technology ownership characteristics of users by health technology use outcomes.

## Abbreviations

ANOVAs: Analysis of variances
CI: Confidence interval
eHEALS: eHealth literary scale
OR: Odds ratio
SF: San Francisco
WIC: Women, Infants, and Children

## References

[1] Greene J, Hibbard JH, Sacks R, Overton V, Parrotta CD (2015). When patient activation levels change, health outcomes and costs change, too. Health Aff (Millwood).

[2] Brown SM, Rozenblum R, Aboumatar H, Fagan MB, Milic M, Lee BS, Turner K, Frosch DL (2015). Defining patient and family engagement in the intensive care unit. Am J Respir Crit Care Med.

[3] Grando M, Grando M, Rozenblum R, Bates D (2015). Information Technology for Patient Empowerment in Healthcare, 1st edition.

[4] Huberty J, Dinkel D, Beets MW, Coleman J (2013). Describing the use of the internet for health, physical activity, and nutrition information in pregnant women. Matern Child Health J.

[5] Amante DJ, Hogan TP, Pagoto SL, English TM, Lapane KL (2015). Access to care and use of the internet to search for health information: results from the US national health interview Survey. J Med Internet Res.

[6] Hostetter M, Klein S, McCarthy D (2014). Commonwealthfund.

[7] Lupton D (2014). Critical perspectives on digital health technologies. Sociology Compass.

[8] Bhandari N, Shi Y, Jung K (2014). Seeking health information online: does limited healthcare access matter?. J Am Med Inform Assoc.

[9] Mackert M, Mabry-Flynn A, Champlin S, Donovan EE, Pounders K (2016). Health literacy and health information technology adoption: the potential for a new digital divide. J Med Internet Res.

[10] Robb M, Shellenbarger T (2014). Himss.

[11] Shieh C, Broome ME, Stump TE (2010). Factors associated with health information-seeking in low-income pregnant women. Women Health.

[12] Fox S, Duggan M (2013). Pew Research Center.

[13] Irizarry T, DeVito DA, Curran CR (2015). Patient portals and patient engagement: a state of the science review. J Med Internet Res.

[14] National Academies of Sciences, Engineering, and Medicine (2016). The Promises and Perils of Digital Strategies in Achieving Health Equity: Workshop Summary.

[15] Lupton D (2016). The use and value of digital media for information about pregnancy and early motherhood: a focus group study. BMC Pregnancy Childbirth.

[16] Wallwiener S, Müller M, Doster A, Laserer W, Reck C, Pauluschke-Fröhlich J, Brucker SY, Wallwiener CW, Wallwiener M (2016). Pregnancy eHealth and mHealth: user proportions and characteristics of pregnant women using Web-based information sources-a cross-sectional study. Arch Gynecol Obstet.

[17] Jang J, Dworkin J, Hessel H (2015). Mothers' use of information and communication technologies for information seeking. Cyberpsychol Behav Soc Netw.

[18] Kraschnewski JL, Chuang CH, Poole ES, Peyton T, Blubaugh I, Pauli J, Feher A, Reddy M (2014). Paging “Dr. Google”: does technology fill the gap created by the prenatal care visit structure? Qualitative focus group study with pregnant women. J Med Internet Res.

[19] Criss S, Woo BJ, Goldman RE, Perkins M, Cunningham C, Taveras EM (2015). The role of health information sources in decision-making among hispanic mothers during their children's first 1000 days of life. Matern Child Health J.

[20] Shieh C, McDaniel A, Ke I (2009). Information-seeking and its predictors in low-income pregnant women. J Midwifery Womens Health.

[21] Bernhardt JM, Felter EM (2004). Online pediatric information seeking among mothers of young children: results from a qualitative study using focus groups. J Med Internet Res.

[22] Norman CD, Skinner HA (2006). eHEALS: the eHealth literacy scale. J Med Internet Res.

[23] Everett A (2008). Learning race and ethnicity: youth and digital media.

[24] DeVellis R (2011). Scale Development: Theory and Applications (Applied Social Research Methods).

[25] Sandefer RH, Westra BL, Khairat SS, Pieczkiewicz DS, Speedie SM (2015). Determinants of consumer eHealth information seeking behavior. AMIA Annu Symp Proc.

[26] Fox S Pew Research Center.

[27] O'Connor S, Hanlon P, O'Donnell CA, Garcia S, Glanville J, Mair FS (2016). Understanding factors affecting patient and public engagement and recruitment to digital health interventions: a systematic review of qualitative studies. BMC Med Inform Decis Mak.

[28] US Department of Health and Human Services, Office of Disease Prevention and Health Promotion Health.

[29] Rock H Rockhealth.

[30] Song FW, West JE, Lundy L, Smith Dahmen N (2012). Women, pregnancy, and health information online. ‎Gend Soc.

