# Supplementary material for: Listening to Communities: Mixed-Method Study of the Engagement of Disadvantaged Mothers and Pregnant Women With Digital Health Technologies
Source: J Med Internet Res. 2017 Jul 5;19(7):e240. doi: 10.2196/jmir.7736 (PMC5517821; doi:10.2196/jmir.7736)
Supplement: Multimedia Appendix 1 [file jmir_v19i7e240_app1.pdf]

Multimedia Appendix 1. Demographic, health, and technology ownership characteristics of users by health technology use outcomes.

			Number of Web-based search activities in last 12 months			Current use of digital health-management practices			Intention to use digital health-management tools		
Characteristic		Total sample (N=92)	<5 (n=33)	5-8 (n=34)	9+ (n=22)	No use (n=39)	Low use (n=28)	High use (n=25)	No interest or low interest (n=22)	Potential adopter (n=45)	High adopter (n=25)
		N (%)	%	%	%	%	%	%	%	%	%
**Childbearing status**											
	Pregnant with no children	26 (28.3)	30.3	38.2	9.1	25.6	28.6	32.0	27.3	26.7	32.0
	Mothers	66 (71.7)	69.7	61.8	90.9	74.4	71.4	68.0	72.7	73.3	68.0
**Location**											
	Bronx	27 (29.4)	39.4	29.4	13.6	35.9	39.3	8.0	27.3	42.2	8.0
	Louisville	16 (17.4)	9.1	8.8	45.5	10.3	3.6	44.0	13.6	4.4	44.0
	SFBA[a]	49 (53.3)	51.5	61.8	40.9	53.9	57.1	48.0	59.1	53.3	48.0
**Age**											
	18-24	23 (25.0)	27.8	17.7	36.4	25.6	21.4	28.0	22.7	24.4	28.0
	25-34	44 (47.8)	45.5	52.9	40.9	38.5	60.7	48.0	54.6	44.4	48.0
	35+	25 (27.2)	27.3	29.4	22.7	35.9	17.9	24.0	22.7	31.1	24.0

			Number of Web-based search activities in last 12 months			Current use of digital health-management practices			Intention to use digital health-management tools		
Characteristic		Total sample (N=92)	<5 (n=33)	5-8 (n=34)	9+ (n=22)	No use (n=39)	Low use (n=28)	High use (n=25)	No interest or low interest (n=22)	Potential adopter (n=45)	High adopter (n=25)
		N (%)	%	%	%	%	%	%	%	%	%
**Race**											
	White	8 (8.7)	6.1	8.8	13.6	5.1	10.7	12.0	9.1	6.7	12.0
	Black	40 (43.5)	45.5	44.1	40.9	35.9	46.4	52.0	36.4	42.2	52.0
	Hispanic or Latina	22 (23.9)	21.2	26.5	22.7	33.3	21.4	12.0	27.3	28.9	12.0
	Asian	15 (16.3)	24.2	11.8	13.6	20.5	14.3	12.0	18.2	17.8	12.0
	Mixed race or other race	7 (7.6)	3.0	8.8	9.1	5.1	7.1	12.0	9.1	4.4	12.0
**Partnership status**											
	Single and not living with a partner	34 (37.0)	30.3	32.4	54.6	29.2	32.1	56.0	36.4	26.7	56.0
	Married or living with a partner	49 (53.3)	60.6	58.8	31.8	61.5	60.7	32.0	45.5	68.9	32.0
	Divorced or separated	9 (9.8)	9.1	8.8	13.6	10.3	7.1	12.0	18.2	4.4	12.0

			Number of Web-based search activities in last 12 months			Current use of digital health-management practices			Intention to use digital health-management tools		
Characteristic		Total sample (N=92)	<5 (n=33)	5-8 (n=34)	9+ (n=22)	No use (n=39)	Low use (n=28)	High use (n=25)	No interest or low interest (n=22)	Potential adopter (n=45)	High adopter (n=25)
		N (%)	%	%	%	%	%	%	%	%	%
**Education**											
	<High school	17 (18.5)	21.2	11.8	22.7	25.6	17.9	8.0	22.7	22.2	8.0
	High school diploma	19 (20.7)	21.2	17.7	22.7	20.5	17.9	24.0	18.2	20.0	24.0
	Some college education	40 (43.5)	48.5	35.3	50.0	46.2	28.6	56.0	45.5	35.6	56.0
	Bachelor's degree or higher	16 (17.4)	9.1	35.3	4.6	7.7	35.7	12.0	13.6	22.2	12.0
**Employment status**											
	Employed	32 (34.8)	36.4	38.2	31.8	33.3	25.0	48.0	50.0	20.0	48.0
	Unemployed	14 (15.2)	21.2	11.8	9.1	20.5	14.3	8.0	13.6	20.0	8.0
	Not in labor force	34 (37.0)	27.3	38.2	45.5	33.3	46.4	32.0	31.8	42.2	32.0
	Student	12 (13.0)	15.2	11.8	13.6	12.8	14.3	12.0	4.6	17.8	12.0

			Number of Web-based search activities in last 12 months			Current use of digital health-management practices			Intention to use digital health-management tools		
Characteristic		Total sample (N=92)	<5 (n=33)	5-8 (n=34)	9+ (n=22)	No use (n=39)	Low use (n=28)	High use (n=25)	No interest or low interest (n=22)	Potential adopter (n=45)	High adopter (n=25)
		N (%)	%	%	%	%	%	%	%	%	%
**Health insurance**											
	Private	7 (7.7)	9.1	12.1	0.0	10.5	3.6	8.0	9.5	6.7	8.0
	Medicaid or public insurance	79 (86.8)	87.9	81.8	95.5	81.6	89.3	92.0	85.7	84.4	92.0
	None	5 (5.5)	3.0	6.1	4.6	7.9	7.1	0.0	4.8	8.9	0.0
**Self-rated health**											
	Excellent	17 (18.5)	21.2	20.6	13.6	10.3	28.6	20.0	18.2	17.8	20.0
	Very good	34 (37.0)	30.3	47.1	31.8	43.6	28.6	36.0	40.9	35.6	36.0
	Good	28 (30.4)	36.4	23.5	31.8	28.2	35.7	28.0	27.3	33.3	28.0
	Fair or poor	3 (14.1)	12.1	8.8	22.7	18.0	7.1	16.0	13.6	13.3	16.0
**Owns or uses a computer**											
	Yes	77 (83.7)	81.8	85.3	81.8	76.9	78.6	100.0	77.3	77.8	100.0
	No	15 (16.3)	18.2	14.7	18.2	23.1	21.4	0.0	22.7	22.2	0.0

			Number of Web-based search activities in last 12 months			Current use of digital health-management practices			Intention to use digital health-management tools		
Characteristic		Total sample (N=92)	<5 (n=33)	5-8 (n=34)	9+ (n=22)	No use (n=39)	Low use (n=28)	High use (n=25)	No interest or low interest (n=22)	Potential adopter (n=45)	High adopter (n=25)
		N (%)	%	%	%	%	%	%	%	%	%
**Owns or uses a smartphone**											
	Yes	80 (87.0)	87.9	91.2	77.3	84.6	78.6	100.0	77.3	84.4	100.0
	No	12 (13.0)	12.1	8.8	22.7	15.4	21.4	0.0	22.7	15.6	0.0

[a]SFBA: San Francisco Bay Area.
